# *De novo* assembly and annotation of *Popillia japonica*’s genome with initial clues to its potential as an invasive pest

**DOI:** 10.1186/s12864-024-10180-x

**Published:** 2024-03-13

**Authors:** Claudio Cucini, Sara Boschi, Rebecca Funari, Elena Cardaioli, Nicola Iannotti, Giovanni Marturano, Francesco Paoli, Mirella Bruttini, Antonio Carapelli, Francesco Frati, Francesco Nardi

**Affiliations:** 1 Department of Life Sciences, University of Siena, Siena, Italy; 2 Council for Agricultural Research and Agricultural Economy Analysis (CREA), Florence, Italy; 3 Department of Medical Biotechnologies, Medical Biotech Hub and Competence Centre, University of Siena, Siena, Italy; 4 Medical Genetics, University of Siena, Siena, Italy; 5 Genetica Medica, Azienda Ospedaliera Universitaria Senese, Siena, Italy; 6 National Biodiversity Future Center (NBFC), Palermo, Italy

**Keywords:** Beetles, Invasive species, Japanese beetle, Odorant receptors, Ionotropic receptors, Gustatory receptors, Cytochrome P450

## Abstract

**Background:**

The spread of *Popillia japonica* in non-native areas (USA, Canada, the Azores islands, Italy and Switzerland) poses a significant threat to agriculture and horticulture, as well as to endemic floral biodiversity, entailing that appropriate control measures must be taken to reduce its density and limit its further spread. In this context, the availability of a high quality genomic sequence for the species is liable to foster basic research on the ecology and evolution of the species, as well as on possible biotechnologically-oriented and genetically-informed control measures.

**Results:**

The genomic sequence presented and described here is an improvement with respect to the available draft sequence in terms of completeness and contiguity, and includes structural and functional annotations. A comparative analysis of gene families of interest, related to the species ecology and potential for polyphagy and adaptability, revealed a contraction of gustatory receptor genes and a paralogous expansion of some subgroups/subfamilies of odorant receptors, ionotropic receptors and cytochrome P450s.

**Conclusions:**

The new genomic sequence as well as the comparative analyses data may provide a clue to explain the staggering invasive potential of the species and may serve to identify targets for potential biotechnological applications aimed at its control.

**Supplementary Information:**

The online version contains supplementary material available at 10.1186/s12864-024-10180-x.

## Background

**Fig. 1:**
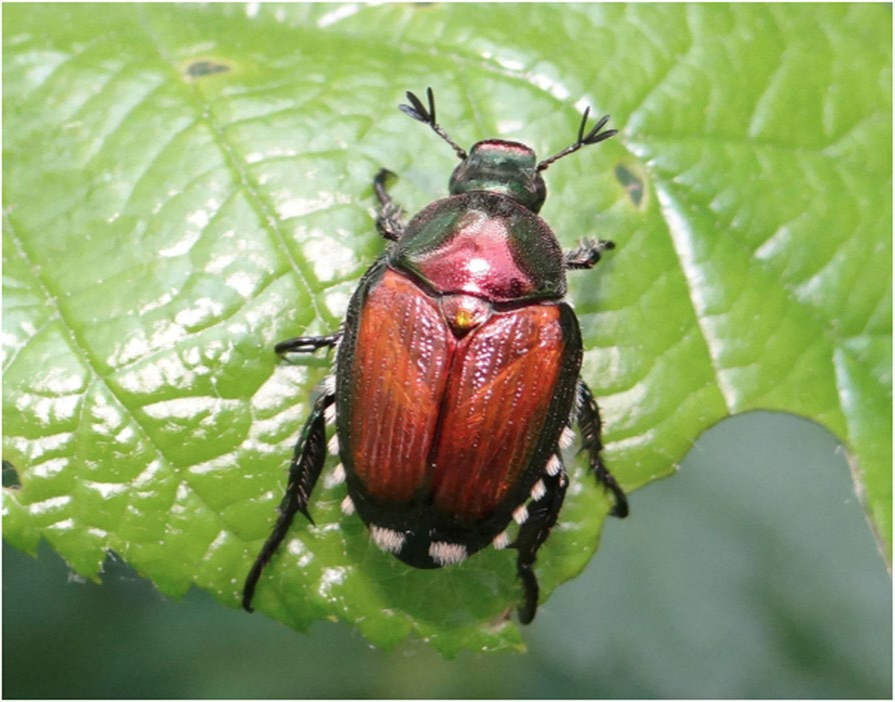
Adult specimen of *P. japonica.* Photo courtesy of Mr. Luciano Gollini

*Popillia japonica* (Fig. 1)*,* commonly known as the Japanese beetle (JB), is a coleopteran species endemic to North-Eastern Asia and Japan [1]. In the last century, it has invaded new areas on two continents, with significant repercussions over the local agriculture as well as the environment. The first appearance beyond its native range in Japan dates back to 1916, when it was recorded in southern New Jersey, USA. JB populations rapidly expanded to colonize the Central/Eastern USA and Canada's Eastern states, further invading the Azores archipelago (Portugal) in the 1970s and mainland Europe, Northern Italy and Ticino (Switzerland), in 2014 and 2017, respectively [2]. This invasion route was recently reconstructed using multiple genetic markers [3, 4]).

*P. japonica* displays a high expansion potential linked to its dispersal ability, with a flight capacity of 12 km per day [5], as well as its prolificacy [6] and the possibility to spread over long distances as a hitchhiker in air cargos (discussed in [7]; [3, 4]). Furthermore, the species displays a high adaptability to different sources of food, being able to feed on over 400 species of plants and vegetables [8] both as larva and adult.

Following its expansion, costs stemming from damages to agriculture and horticulture, as well as costs for monitoring and control, are rapidly increasing. It has been estimated that more than $460 million per year [9] are needed to control the spread of the species in the US. Furthermore, a recent projection on potential damages in the European Union (EU) led to estimates from €30 million to €7.8 billion per year [10]. As such, *P. japonica* is creating the utmost concerns and has been included in the list of quarantine pest by the European Plant Protection Organization.

In response to the challenges posed by this pest, diverse strategies have emerged in a bid to curtail its advance and limit population density: (a) biological control (e.g., natural predators, entomopathogenic nematodes and fungi); (b) chemical control (e.g., ɑ-cypermethrin, deltamethrin); (c) physical control (e.g., attract-and-kill devices or nets) (recently reviewed in [2] and [6]). Nevertheless, the potential for biotechnological applications to control the pest, or the integration of molecular genetic information to steer the advancement of non-biotechnological approaches, has been hindered, with notable exceptions [11], by the lack of a high quality, annotated, genome for the species. As a term of comparison, among the 20 quarantine pests, predominantly insects, categorized as high-priority targets for the EU in 2019, approximately 60% have one or more genome sequences available, 25% have an ongoing genome project, while a mere 15% seem to lack genomic data altogether (data compiled from https://www.ncbi.nlm.nih.gov/datasets/genome/).

Early attempts to generate genomic data for the JB include transcriptome sequencing at the University of Southern Mississippi in 2013, followed by genome sequencing by Iridian Genomes in 2016. The current reference genome for the species was produced by Canada’s Genomic Enterprise (CGE), within the framework of the CanSeq150 initiative, and was released in 2019 under BioProject PRJNA514809 (NCBI). The sequence has been obtained from a male specimen collected in Vancouver, a representative of the North American invasive population [3]. However, this sequence has some limitations, namely: a) it has been assembled using Illumina short reads only; b) it does not include structural or functional annotations; and c) it has not been formally documented and described in a published paper.

Among the most frequently studied gene families linked to insect ecology (e.g. feeding, chemical interactions, sensory functions) are gustatory receptors (GRs), odorant receptors (ORs) and ionotropic receptors (IRs), that together process olfactory information, as well as cytochrome P450 genes (CYP) which are implied in a wide variety of functions as compound detoxification and hormone metabolism. While not a simple linear relationship, some authors pointed out that these genes, and possible expansions/contractions of these gene families, might be associated to some important aspects of a species’ ecology, including polyphagy, life style and adaptability [12–15]. Notable examples of this association might be the OR arsenal in coleopterans, which greatly diverge between Adephaga and Polyphaga species [15], or the expansion of the CYP repertoire in the polyphagous mite *Tetranychus urticae,* which is doubled compared to the congeneric oligophagous *T. evansi* and the monophagous *T. linteariuse* [14]. As such, an in-depth characterization of the species’ genetic arsenal is of primary interest for understanding the JB invasive and damaging potential.

Gustatory receptors are the primeval chemosensory receptors in Arthropoda and are expressed in various tissues. They are divided in several subfamilies, some of which have been associated with the chemoreception of specific compounds such as carbon dioxide, fructose and sugar [16, 17]. Notwithstanding a substantial residual diversity in the family, described in previous works, their functional characterization has been proven mainly in dipterans and lepidopterans [17, 18] while in non-model coleopterans their functions are only indirectly inferred.

Odorant receptors are primarily expressed in olfactory sensory neurons (OSNs) of antennae and maxillary palps and are involved in the detection of volatile compounds [16, 19]. ORs were recently categorized in nine groups *plus* Orco [15]. A single conserved copy of *orco* is present in all Dicondylia (Zygentoma + Pterygota), alongside a variable number of OR genes [17, 20]. Each OR, by binding to the co-receptor Orco to form a ligand-gated ion channel, is able to detect one single odorant molecule. Therefore, the number of different OR genes can be regarded as a proxy for a species capacity to recognize different volatiles [20].

Ionotropic receptors evolved from the conserved family of ionotropic glutamate receptors (iGluRs) at the base of the Protostomia clade [16, 17] and are characterized by two extracellular ligand-binding domains plus three transmembrane domains. They are divided in two main groups: antennal IRs, expressed in antennal neurons, and divergent IRs, expressed in other tissues. While the functional role of different IRs has not been characterized in detail, the former group seem to be involved in the recognition of acids, ammines and amino acids [17].

Cytochrome P450 genes encode for a wide array of monooxygenases that are involved in different biological processes, from ecdysteroid metabolism to foreign compounds transformation, as well as insecticide resistance [14, 21]. CYP genes can vary in length, abundance and function, but they typically share a conserved central active site. Arthropod CYP genes were recently systematized as belonging to six clans (CYP2, CYP3, CYP4, CYP16, CYP20 and the mitochondrial clan), with CYP16 and CYP20 generally missing in insects [14].

In this study, the genome of an individual of *P. japonica* sampled in Northern Italy, close to the site of the original Italian introduction, is described. A special attention was devoted to four gene families of interest (i.e., ORs, ORs, IRs and CYP genes) as liable to shed light over the staggering adaptability, polyphagy and invasive potential of the species.

## Methods

### Sampling, extraction and sequencing

Individuals of *P. japonica* were collected during summer 2020 and 2021 in the outskirts of Cameri (NO, Italy, 45.5128460N 8.6908590E) at a distance of ~ 4 km from the site of the first Italian report in 2014 (Naviglio Vecchio, Turbigo, MI, Italy; [22]). Additional samples for RNA sequencing were collected from the neighboring locations of Novara (NO, Italy, 45.44316N 8.66766E) and Briona (NO, Italy, 45.541974N 8.531064E). Samples were frozen in liquid nitrogen and stored a -80 °C until processing. Notably, these individuals come from one population characterized by extremely reduced genetic variability [3], therefore limiting the drawbacks of using multiple individuals for genome assembly.

Males, the heterogametic sex [23], were chosen for genome sequencing. High molecular weight DNA was obtained from dissected male testes, to minimize foreign contaminants, using the Wizard® Genomic DNA Purification Kit following the manufacturer’s specifications. Three males (identified as DMR4, DMR5 and DMR6 in NCBI) were individually processed to produce three libraries with the Illumina DNA Prep Tagmentation kit. Libraries were sequenced, with a 250 bp PE layout, employing a S4 flow cell on a NovaSeq6000 machine at the Department of Medical Biotechnologies of the University of Siena. Two libraries, from one male (DMR5) and a pool of three males (collectively identified as DMR28), were used for long read sequencing using the Ligation sequencing kit and sequenced using a FLO-MIN106 flow cell on an Oxford Nanopore MiniION machine at Bio-Fab Research (Rome).

Total RNA for transcriptome sequencing was purified from the whole body of single individuals using the QIAGEN RNeasy Micro kit, inclusive of QIAshredder and DNAse treatments. Two replicates for each of four different sexes/stages (larvae, pupae, adult males and adult females) were used to increase representativeness and diversity in the transcriptomic output. Libraries were prepared using the TruSeq Stranded Total RNA with Ribo-Zero kit for total RNA sequencing and TruSeq stranded mRNA kit for mRNA sequencing. The 16 libraries were sequenced with a 150 bp PE layout using a NovaSeq6000 machine at Macrogen (Amsterdam). Samples were RL11 and RL14 (larvae), RP6 and RP15 (pupae), RF9 and RF15 (adult females), RM4 and RM8 (adult males) for both total and mRNA sequencing.

### Sequence pre-processing

Illumina raw reads were checked in FastQC (v. 0.11.8; [24]) and trimmed using cutadapt (v. 3.4; [25]) and trimmomatic (v. 0.33; [26]) under default settings. Nanopore raw reads were checked as above and trimmed using NanoFilt (v. 2.8.0; [27]) with a minimum quality of 8 and a minimum read length of 1500 bp. Sequences were preliminarily mapped on the mitochondrial genome available in NCBI (NC_038115) using minimap2 (v. 2.24; [28]) and mapping reads, of mitochondrial origin, were removed prior to the assembly.

### Assembly

The three Illumina DNA libraries were individually assembled using a *de novo* approach in ABySS (v. 2.3.7; [29]) with default settings and *k* = 96. The two Nanopore libraries were merged to increase read count and assembled *de novo* using Flye (v. 2.9.1; [30]) with six rounds of polishing iterations (leading to a stabilization of the error rate). The four assemblies were combined in MAC (v. 2.0; [31]) to generate a single high quality merged product. Redundancy and contaminants were removed from the assembly via purge_haplotigs (*low* = *25 mid* = *150 high* = *275*, v. 1.1.2; [32]) producing a primary draft assembly. All RNA sequencing data were remapped on the draft assembly using HISAT2 (v. 2.2.1; [33]) and P_RNA_scaffolder [34] was used for scaffolding. A second round of purge_haplotigs (*low* = *18 mid* = *153 high* = *279,* v. 1.1.2) was performed to remove any duplication/contaminant that may have become visible during the scaffolding step. Foreign Contamination Screen (v. 0.3.0; [35]) was used to identify and remove any additional contaminant sequences (bacteria, fungi, protists, viruses) and technical sequences from the primary assembly, obtaining the final assembled genome.

### Annotation

A custom library of repeated elements was produced by scanning the final assembly with RepeatModeler (v. 2.0.2a; [36]). The model was then used in RepeatMasker (v. 4.1.2-p1; [37]) for soft masking of repeated elements and low complexity regions in the assembly. Sequence data from mRNA libraries were remapped over the genomic sequence using HISAT2 and structural annotation was accomplished using BRAKER (v. 2.1.6, pipeline B, RNA-Seq guided; [38]). Functional annotation was performed using InterProScan (release 5.60–92.0) and Pfam, InterPro and Ontology terms were annotated on transcripts. The final masked genomic sequence, inclusive of structural and functional annotations, was deposited in GenBank under accession number JASPKY000000000.

### Genome completeness and statistics

Assembly statistics (N50, number of scaffolds, remapped reads count, etc.) were estimated and compared between the Italian and Canadian genome of *P. japonica* trough QUAST-LG (v. 5.0.2; [39]). Due to the absence of a true chromosome level reference, the Italian and the Canadian genomes were in turn used as reference into two parallel analyses. Assembly accuracy was assessed trough a k-mer spectrum analysis in Merqury (v. 1.3 [40]) using Illumina reads (DMR4-6). To evaluate the quality of the annotation, trimmed RNA-seq reads were mapped back to the annotation with HISAT2 (v. 2.2.1; [33]) and relative metrics were extracted and visualized from the BAM file trough samtools (*view, stat*) (v. 1.13; [41]). Moreover, a BUSCO analysis was performed to evaluate the completeness at the annotation level (v. 5.4.4, Endopterygota lineage; [42]). Results were visualized with *ggplot* in the R environment.

Annotation statistics (number of genes/transcripts, number of introns/exons) were produced using in-house python scripts employing the *GFFExaminer* and *pandas* packages.

Completeness, fragmentation and duplication level of the newly assembled genome, alongside all Coleoptera RefSeq genomes available in March 2023 in NCBI, *Apis mellifera* (GCF_003254395.2) and the *P. japonica* genome produced by the CanSeq150 project (GCA_004785975.1), were assessed using BUSCO (v. 5.4.4, Endopterygota lineage; [42]).

Single-copy orthologs of the Endopterygota collection (*n* = 2,124) were extracted from the aforementioned genomes using BUSCO, individually aligned with mafft (v. 7.475 using the *–auto* parameter; [43]) and quality-checked with AMAS (Alignment manipulation and summary statistics; [44]). High quality alignments (i.e., < 10% of missing info) were retained and combined in a single matrix. A phylogenomic tree was constructed using IQTREE2 (*-B 2000 -m MFP*, v. 2.2.0 [45];). The final tree was visualized and edited using the *ggtree* library in R (v. 3.8.2; [46]).

### Comparative analyses

Pre-built HMM profiles characteristic of GRs, IRs, ORs and cytochromes P450 were downloaded from Pfam as in [47] and [48]. In detail, Pfam domains PF06151 (trehalose receptor) and PF08395 (7tm chemosensory receptor) were used to identify GRs. Pfam domains PF00060 (an extracellular domain, also known as S1 domain) and PF10613 (Lig_chan transmembrane domain) were used to determine IRs. Pfam domain PF02949 (7tm receptor) was used to find ORs. Pfam domain PF00067 (heme-binding loop, helix groove and the conserved EXXR motif) was used to identify CYP genes.

Pfam profiles were pressed *(hmmpress*) and scanned (*hmmscan*) against the *P. japonica*’s translated CDS (including spliced variants) collection using HMMER (v. 3.3.2; [49]), applying an E-value threshold of 1e-05, to identify genes pertaining to the aforementioned families. For those groups were more than one Pfam domain was used (i.e., GRs and IRs), the arising datasets were combined and only unique entries were retained. To further filter out possible spurious matches, each resulting sequence was compared to the nr database (NCBI) using BLASTp [50] and only sequences whose best hit matched with the expected family were retained.

The resulting sequences were combined with the most recent curated datasets of beetles as follows. For GRs and IRs four species from [19, 51, 52] were included (*Dendroctonus ponderosae, Agrilus planipennis, Leptinotarsa decemlineata, Tribolium castaneum*). For ORs three species from [15] were included (*Anoplophora glabripennis*, *L. decemlineata and T. castaneum*). For cytochromes P450 three species from [14] were included (*D. ponderosae*, *Nicrophorus vespilloides* and *T. castaneum*). The four datasets were independently aligned in MUSCLE (v. 3.8.425; [53]), trimmed in trimAl (*-gt 0.7 -st 0 -cons 25* as in [19] and [15], v. 1.2rev59; [54]) and manually curated in Aliview (v. 1.28; [55]). Curated datasets were used to infer the phylogeny of the four gene families in IQTREE2, with automatic model selection and 2,000 replicates of UFB and SH-aLRT. Final trees were visualized in iTOL (v. 6; [56]) and customized using the *itol.toolkit* R package [57]. Trees were annotated based on gene family and subfamily annotations from previously published works (see Results).

Gene counts at the gene family and subfamily levels were determined based on individual genes (i.e. excluding splice variants). This conservative position was taken to avoid potential biases associated the presence of splicing variants, hence facilitating the comparison of gene family expansion across species.

## Results

### Genome sequencing, assembly and annotation

DNA Illumina sequencing produced between 333.2 and 383.5 million read pairs in the three libraries. ONT long read sequencing produced a total of 1.8 million reads. Based on an expected genome size of ~ 0.6 Gb, these correspond to a theoretical coverage of ~ 300 × for Illumina (effective coverage ranging from 206 × to 226x) and 20x (effective coverage of 18x) for ONT long reads. Messenger RNA sequencing produced between 41.0 and 52.0 million reads pairs in 8 libraries, total RNA sequencing between 20.6 and 21.2 million read pairs in 8 libraries.

**Fig. 2:**
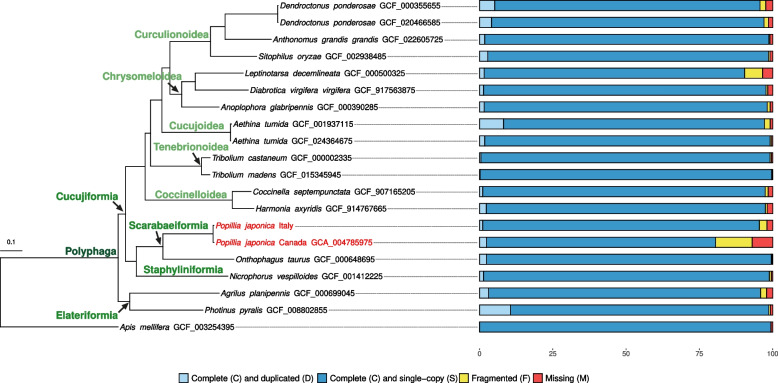
Phylogenetic relationships among Coleoptera based on high-quality aligned single-copy orthologs derived from the BUSCO analysis. All the nodes have a bootstrap value of 1. Each species is coupled with the corresponding BUSCO graphic. The two available genomes of *P. japonica* (from the CanSeq150 project as well as this study) are highlighted in red. Higher ranking categories are indicated at relevant nodes with green shades

The final polished assembly was 578.347 Mb in length and was fragmented in 6,164 scaffolds with a N50 of 0.895 Mb (Tab. S1). The k-mer spectrum plot identified a monomodal distribution of k-mers over the assembly with a single major peak, confirming that one single haplotig was retained (Fig. S1). BUSCO statistics resulted as 94.3% of complete BUSCOs, 1.1% of duplicated BUSCOs, 2.6% of fragmented BUSCOs and 2.0% of missing BUSCOs (Fig. 2, Tab. S2).

A comparison of the new *P. japonica* assembly with the previously available genome from the CanSeq150 program (NCBI GCA_004785975) shows that the new genome is slightly longer (578 Mb *vs.* 531 Mb), more complete (94.3% *vs.* 78.1%), less duplicated (1.1% *vs.* 2.4%), largely less fragmented (2.6% *vs.* 12.5%), as well as more contiguous (6,164 scaffolds *vs.* 75,144; N50 = 0.895 Mb *vs.* 0.015 Mb; Tab. S1, S2, Fig. S2). A comparison of BUSCO statistics with all available RefSeq genomes of Coleoptera (Fig. 2) places the new sequence among the lowest in terms of duplication and well in the range of other RefSeq genomes in terms of completeness and fragmentation.

The phylogenetic analysis, based on a supermatrix of 1,437,041 amino acid positions by 19 coleopteran taxa *plus Apis mellifera* as outgroup, produced the tree represented in Fig. 2. Overall, phylogenetic relationships are in line with the currently accepted taxonomy of the group. The new genome is recovered, alongside the previously available JB genome, as the sister group of *Onthophagus taurus* within Scarabeiformia (Scarabaeidae) (Fig. 2).

**Fig. 3:**
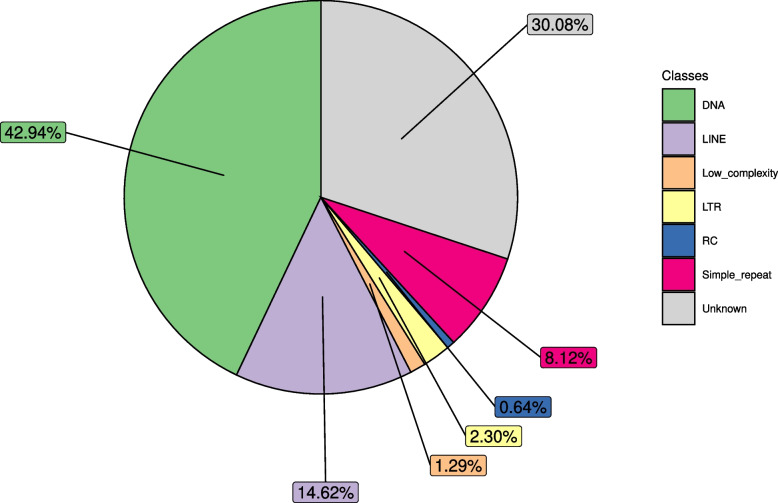
Relative abundance, as percentages, of transposable elements in the *P. japonica’*s genome. TE classes are based on RepeatMasker annotation. Despite the high quantity of unclassified repetitive regions, the vast majority (~ 57.56%) is represented by DNA elements and LINEs. Classes are color coded

A large portion of the genome (36.7%) is composed of interspersed repeats and low complexity regions. Among these, the most abundant are DNA elements (42.9%), Long Interspersed Nuclear Elements (LINEs) (14.6%) and simple repeats (8.1%), whereas Long Terminal Repeats (LTRs), low complexity regions and Rolling Circles (RCs) were observed in lower proportions (Fig. 3, Fig. S3). The most frequent repeats are TcMariner transposons (with *TcMar-Tc1* accounting for ~ 34% of all annotated TEs; Fig. S3). Notwithstanding the presence of numerous outliers characterized by a large dimension (up to 6 Kb in DNA elements), the vast majority of TEs are short elements with an average length of ~ 200 bp (Fig. S4). This feature might represent the vestigial trait of remnant TEs which underwent a loss of their distinctive domains.

Automatic structural annotation identified 31,668 genes transcribed as 36,495 different transcripts (splice variants), with the vast majority of genes (89.1%) being transcribed as a single transcript. BUSCO Endopterygota assessment on single-genes at the annotation level highlighted a high degree of completeness with a small fraction of duplication (Fig. S5). The distribution of the number of exons per transcript (Fig. S6) is shaped as a quickly sloping curve, with most genes consisting of a single (27.9%) or few (≤ 5; 74.2%) exons. Functional annotation analysis produced a viable annotation for 18,725 transcripts (51.3%) in at least one database. Namely, all the annotated transcripts have received a Pfam annotation (51.3% of total transcripts), 12,228 (33.5%) an InterPro annotation and 10,668 (29.2%) a Gene Ontology term. A total of 14,958 genes have at least one transcript with associated functional annotation in one or more databases.

### Gustatory receptors

The analysis of GRs in *P. japonica* identified a total of 53 putative GRs encoded by 51 different genes *plus* two splice variants. Among the species taken into account, the number of GR genes in the JB genome is in line with *A. planipennis* (30) and *D. ponderosae* (49) but far lesser than in *L. decemlineata* (90) and *T. castaneum* (183) (Tab. 1).

**Table 1:** Putative GR gene counts (excluding splicing variants) in *P. japonica* and other beetle species The first column indicates the total number of gustatory receptor genes in the different species, * after [19] dataset, ** after [52] dataset, *** after [51] dataset. The last three columns report gene counts in specific gene subfamilies

Species	Total GRs	Fructose GRs	CO_2_ GRs	Sugar GRs
A. planipennis*	30	1	2	6
D. ponderosae*	49	1	3	6
L. decemlineata**	90	1	3	6
*P. japonica*	51	0	2	3
T. castaneum***	183	8	3	16

The first column indicates the total number of gustatory receptor genes in the different species, * after [19] dataset, ** after [52] dataset, *** after [51] dataset. The last three columns report gene counts in specific gene subfamilies

**Fig. 4:**
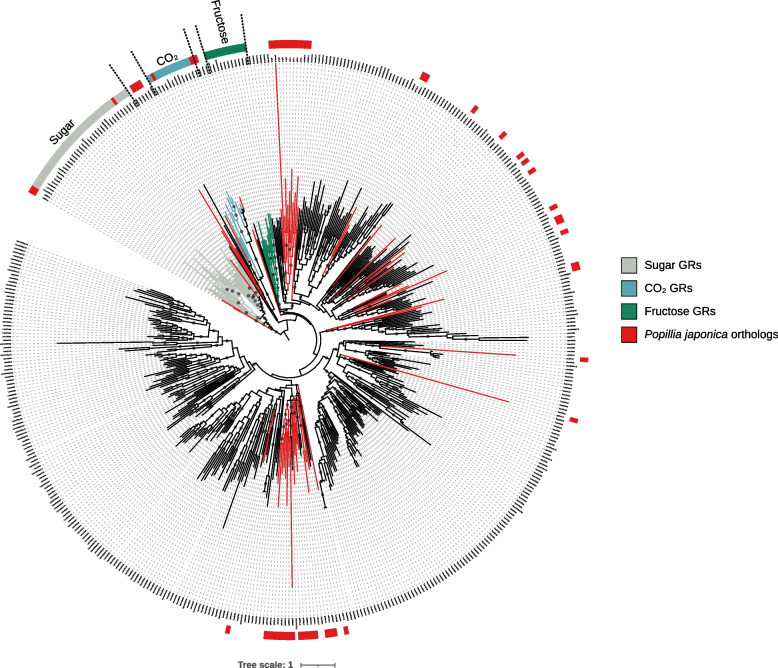
Phylogenetic reconstruction of gustatory receptors (including splicing isoforms) observed in the genome of *P. japonica* as well as *T. castaneum, L. decemlineata, D. ponderosae* and *A. planipennis.* Relevant clades are color coded. The large number of non-annotated branches reflects current limitations in knowledge of this receptors. Putative orthologs in *P. japonica* are highlighted in red. The tree is rooted with the clade of conserved sugar GRs. Supported nodes (SH-aLRT >  = 70, UFB >  = 85) are identified with a dark gray dots along the branch

Based on the subfamily-level annotation of genes from the 4 species selected for comparison, and following phylogenetic analysis, the three conserved clusters corresponding to sugar, carbon dioxide and fructose receptors were identified (Fig. 4). Despite the generally conserved presence of 3 GR genes for carbon dioxide in most species, only 2 were identified in the JB. Within the sugar clade, only 3 GRs were identified in the JB, whereas other species are characterized by six or more. Moreover, the JB genome is apparently missing fructose receptors altogether, whereas other beetles have at least one. Worth of note, these three subfamilies account for a marginal fraction of the overall, not functionally characterized, diversity of GR genes in all species. Within these divergent GRs, *P. japonica* is characterized by few ortholog copies while a marked paralogy is evident in multiple branches (Fig. 4).

### Odorant receptors

**Fig. 5:**
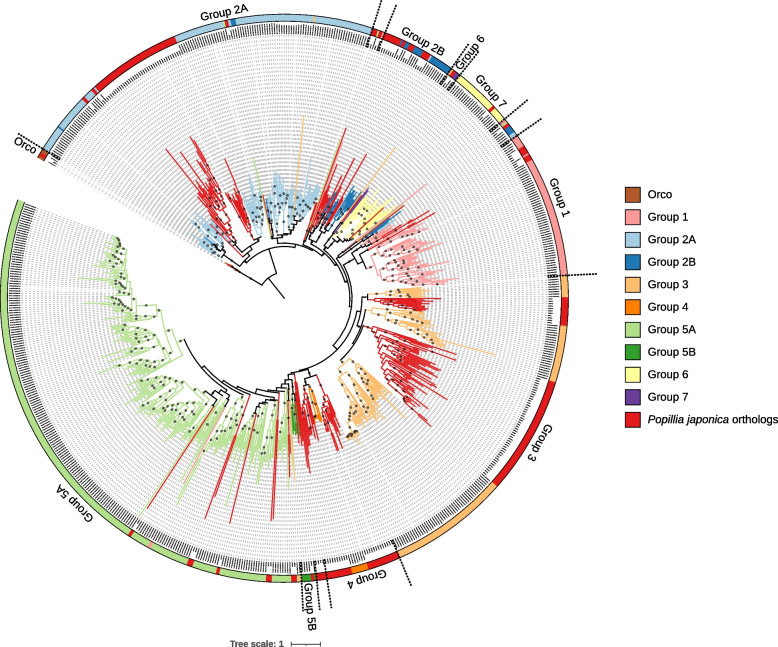
Phylogenetic reconstruction of odorant receptors (including splicing isoforms) in the genome of *P. japonica* as well as *A. glabripennis*, *L. decemlineata* and *T. castaneum*. Relevant clades, following the classification in [15], are color coded. Putative orthologs in *P. japonica* are highlighted in red. Eighteen JB outliers were not recovered within orthologous clades and hence considered as outliers. The tree is rooted on the conserved Orco group. Supported nodes (SH-aLRT >  = 70, UFB >  = 85) are identified with a dark gray dot along the branch

A total of 159 ORs, encoded by 149 different genes, were identified in the genome of *P. japonica*. Based on the phylogenetic reconstruction*,* and leveraging on the recent classification of ORs proposed by [15], most JB ORs could be unequivocally classified in the canonical 9 subfamilies (Group 1, 2A, 2B, 3, 4, 5A, 5B, 6, 7 and Orco; Fig. 5). Eight ORs did not cluster within the described groups and were retrieved as sister sequences of the major subfamilies. Moreover, a small fraction of JB ORs (18 out of 522) did not cluster within the previously described clades and were identified as outliers. After rooting on the most conserved Orco clade, as the most conserved group, Group 2A was recovered as paraphyletic in a basal position with respect to other subfamilies, that are all recovered as a single monophyletic and well supported clade (Fig. 5).

**Fig. 6:**
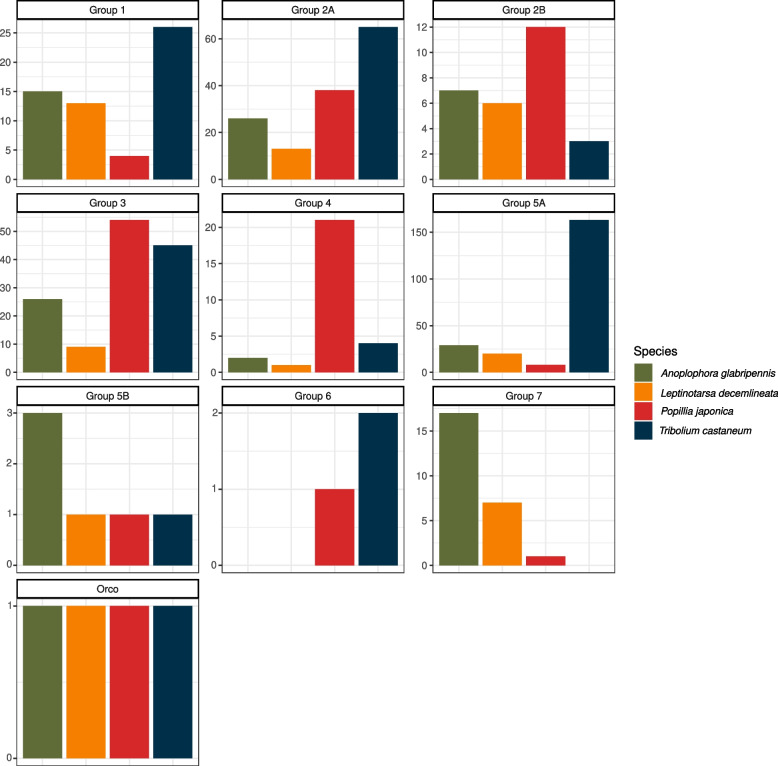
OR gene counts (excluding splicing variants) in the genome of *P. japonica* as well as *A. glabripennis*, *L. decemlineata* and *T. castaneum*. Subfamilies were defined following the phylogenetic reconstruction of ORs (Fig. 5). Group 2B, 3 and 4 show a strong expansion in *P. japonica’*s genome (from 1.2x to 21x) in comparison to other species. Instead, Group 1 and 5A apparently experienced a gene contraction in JB genome. Species are color-coded

The JB shows at least one representative OR in all the studied clades. Groups 5B, 6, 7 and Orco were distinguished by a single ortholog while multiple ORs were identified for other groups. A significant paralogous expansion was identified in Groups 2B, 3 and 4, where the number of hypothetical JB ORs is 1.2 × to 21 × in comparison with the other species studied (Fig. 6). In turn, Groups 1, 5A and 7 appear less represented in the JB.

### Ionotropic receptors

**Fig. 7:**
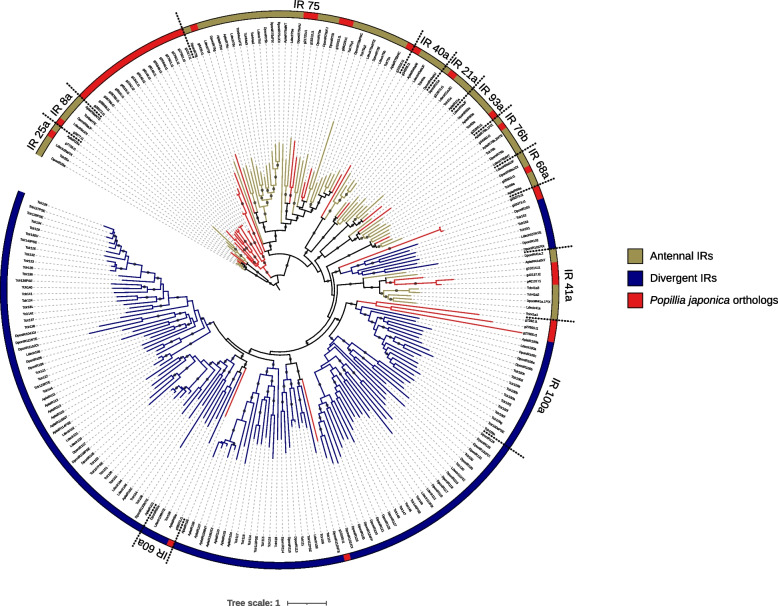
Phylogenetic reconstruction of ionotropic receptors (including splicing isoforms) in the genome of *P. japonica,* as well as *T. castaneum, L. decemlineata, D. ponderosae, A. planipennis*. The two annotated clades (antennal and divergent) are color coded. Putative orthologs in *P. japonica* are highlighted in red. Fine scale subfamily level annotation of groups is provided in the outer most circle. The different density of subfamilies level annotation reflects the current limited knowledge of gene divergent IR subfamilies. The tree is rooted on the conserved IR8a and IR25a clades. Supported nodes (SH-aLRT >  = 70, UFB >  = 85) are shown with a dark gray dot along the branch

A total of 40 IRs, encoded by 31 different genes, were identified in the genome of *P. japonica*. Based on the phylogenetic reconstruction (Fig. 7), as well as subfamily-level annotations available for *T. castaneum, L. decemlineata, D. ponderosae* and *A. planipennis* IRs, the JB’s IR repertoire could be assigned to both antennal (IR8a, IR21a, IR25a, IR40a, IR41a, IR68a, IR75, IR76b and IR93a) and “divergent” (IR60a, IR100a and other non-classified IRs) clades. The most conserved subfamilies (antennal, IR60a, IR100a) were recovered as monophyletic in the tree, whereas divergent IRs, less characterized, were recovered as polyphyletic due to the placement of a small group of sequences among antennal IRs. Interestingly, a small group of the JB IR sequences that form a monophyletic clade was identified near the base of the tree. These sequences could not be annotated due to the absence of a known ortholog in the group but, given their position in the tree they can be tentatively regarded as antennal and are hereafter referred to as “JB IR basal group”.

**Fig. 8:**
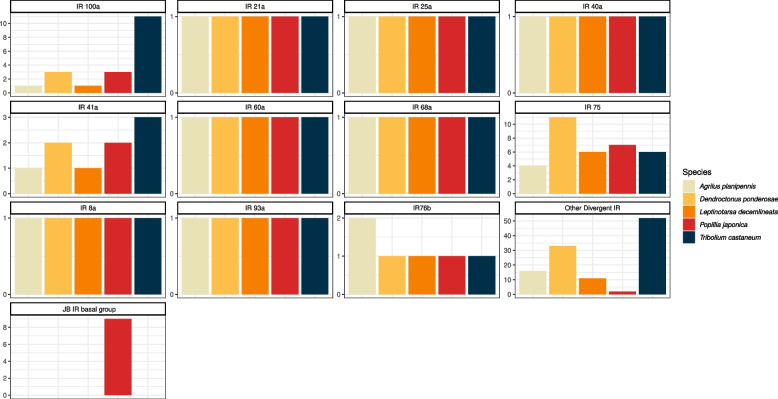
IR gene counts (excluding splicing variants) in the genome of *P. japonica* as well as *T. castaneum, L. decemlineata, D. ponderosae, A. planipennis*. Subfamilies were defined following the phylogenetic reconstruction of IRs (Fig. 7). IRs which did not belong to any family were cataloged as “divergent IRs”, while the well clustered monophyletic paralogous clade of *P. japonica* is classified as “JB IR basal group”. Species are color-coded

In terms of the diversity within individual IR subfamilies, the number of IR genes identified in the JB is grossly in line with other species. Subfamilies IR8a, IR21a, IR25a, IR40a, IR60a, IR68a, IR76b and IR93a are represented by one single sequence. IR41a, IR75, IR100a, as well as other divergent IRs, are represented by multiple paralogs (Fig. 8). Facing a contraction of other divergent IRs, that are nevertheless a diverse and not well characterized assemblage, an apparently new clade was identified among antennal IRs.

### CYP genes

**Fig. 9:**
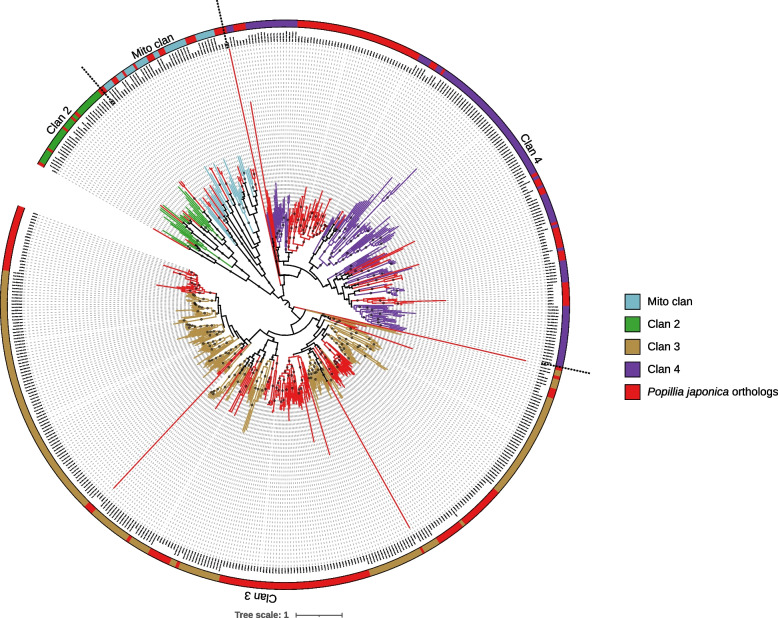
Phylogenetic reconstruction of CYP 450 (including splicing isoforms) in the genome of *P. japonica* as well as *D. ponderosae*, *N. vespilloides* and *T. castaneum*. Clans are color coded following [14]. Putative orthologs in *P. japonica* are highlighted in red. All CYP clans were found as monophyletic clades. The tree is mid-point rooted. Supported nodes (SH-aLRT >  = 70, UFB >  = 85) are identified with a dark gray dot along the branch

A total of 198 cytochromes P450, encoded by 158 different genes, were identified in the genome of *P. japonica*. Clan-level annotation of individual sequences was accomplished based on the phylogenetic reconstruction (Fig. 9) leveraging on annotations of *D. ponderosae*, *N. vespilloides* and *T. castaneum* [14]. All four canonical CYP clans (clan 2, clan 3, clan 4 and mito clan) are recovered as monophyletic clusters in the phylogenetic tree (Fig. 9). Clan 3 emerges as the most divergent one, and clan 4 as the sister group to the remaining clans (mito and 2).

**Fig. 10:**
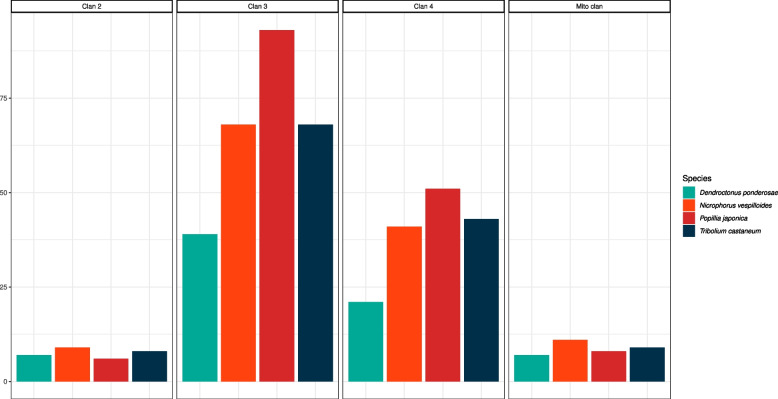
CYP 450 gene counts (excluding splicing variants) in the genome of *P. japonica* as well as *D. ponderosae*, *N. vespilloides* and *T. castaneum*. Subfamilies were defined following the phylogenetic reconstruction of cytochrome P450 (Fig. 9). An apparent gene expansion is retrieved in *P. japonica’*s clan 3 and 4. On the contrary, clan 2 and mito clan gene number of the JB are in line with other coleopteran species. Species are color-coded

Concerning the diversity within individual clans, the mito clan and clan 2 are relatively small, with 8 and 6 JB orthologs, respectively, and their numerousness is in line with other species under scrutiny (Fig. 10). On the other hand, an expansion was observed in clan 3 and clan 4. While the number of JB CYPs belonging to these two clans outnumbers those observed in other species, namely *N. vespilloides* and *T. castaneum*, by a mere ~ 1.2x and 1.3x respectively, the presence of 2–4 large monophyletic groups of JB CYPs within clan 4 and especially clan 3 suggests that specific CYP orthologs may have underwent a significant paralogous expansion in the JB’s genome.

## Discussion

Despite the availability of a draft sequence of the *P. japonica* genome since 2019, and the sheer interest raised by this pest in its expansion within the USA and to Europe, studies on the species, as on its biology and possible methods of control, did not leverage in full on available genetic data. In fact, molecular genetic applications, despite some notable attempts, are still limited. The new assembly presented here improves over the available draft in terms of completeness and contiguity, and includes a structural and functional annotation. This information is liable to shed light at the molecular level on several processes such as the extent of natural selection during the invasion process, or the modifications to gene expression connected to the response of the pest to different control measures. Studies on these issues are ongoing. In addition, the raw data produced and publicly available (Illumina and long ONT DNA reads, mRNA and total RNA reads from different life stages) may constitute a solid basis for future research on this pest.

While the primary aim of this study was not to resolve the conflicts that often arise in coleopteran systematics, the phylogenomic tree presented as Fig. 2 is a useful contribution in this direction. In fact, BUSCO single-copy orthologs have been shown to be a powerful tool to investigate phylogenetic relationships at the whole genome level [58]. Notwithstanding taxon sampling, limited to the 16 different species whose complete genome is available, the phylogenomic reconstruction supports, with high confidence, the previously proposed framework of Coleoptera systematics proposed by [59].

Concerning genome size and the repertoire of transposable elements, it has been suggested that, yet in a context of large variability, the amount/size of TEs is a major driver of genome size evolution in eukaryotes [60]. As an example, insects characterized by a small genome (e.g., the midge *Belgica antarctica,* 0.89 Mb) tend to have a limited repertoire of TEs (2.5% in the species), while insects with an extremely large genome (e.g., the grasshopper *Locusta migratoria*, 5.75 Gb) have an expanded content of TEs (63.5%). Excluding extreme cases, and focusing on insect species with a regular (from 0.1 Gb to 2.7 Gb) genome size, the TE content is in the range of 17% to 30%. In Coleoptera, with genomes in the range of 0.19 Gb to 1.1 Gb TE content is 12.9–41.4%. In this context, the JB shows a sizable, though not extreme, expansion of the TE repertoire (36.7%), ranking 13th out of 74 insect species studied by [60] plus *N. vespilloides* [61] and 2nd out of the 6 coleopterans (Fig. S7). The relative abundance of different classes of TEs (Fig. 3, Fig. S7) is grossly in line with other coleopterans studied by [60] and [61], with DNA elements and LINEs being the most represented classes of TEs.

In order to find genetic-based clues to the staggering potential of *P. japonica* as an invasive pest (i.e., polyphagy, adaptability, resistance), four gene families (GRs, ORs, IRs, CYPs) were studied in detail to characterize the JB genetic repertoire connected to sensing and detoxification. Given that these analyses rely on annotated genes, the quality of the annotation itself is a critical prerequisite. The remapping rate over the transcriptome was found to be acceptable but not particularly high (~ 57%, Tab. S3). This may stem from annotating only coding sequences (CDS) without including 5' and 3' untranslated regions (UTRs), or from overlooking transcribed genes with low expression levels or those specific to certain stages or tissues. The number of annotated genes, while relatively high compared to closely related species such as *Cetonia aurata* and *Tripoxylus dichotomus* [62, 63], generally aligns with expectations within the larger context of Coleoptera (e.g. *Gonioctena quinquepunctata*, *Acanthoscelides obtectus*, and *Callosobruchus chinensis;* Genome-NCBI https://www.ncbi.nlm.nih.gov/genome/). It's worth noting that the annotation may have limitations, such as incomplete coverage or inaccuracies, and more specifically stems from an automatic procedure and does not include a thorough manual curation at this stage. Nevertheless, and despite these potential weaknesses, it's important to highlight that the BUSCO duplication rate at the annotation level (i.e. excluding splice variants) was observed to be very low (Fig. S5). Consequently, any biases in gene family counts are likely conservative, which strengthens our confidence in discussing gene copy amplification.

Odorant receptors are encoded by 149 genes, few of them expressed as multiple transcriptional variants (Figs. 5, 6). Orthologs were identified for each of the canonical subfamilies and, to our best knowledge, *P. japonica* shows one of the most extensive repertoire of odorant receptors, ever described among beetles even excluding transcriptional isoforms. The species has one copy of each of Group 6 and Group 7 orthologs, which are the two less conserved Groups among Polyphaga (Fig. 6). In fact, Group 6 is missing in both Chrysomelidae and Curculionidea while Group 7 is missing in Elateriformia and Tenebrionoidea, perhaps entailing two independent gene losses [15]. Similarly, also *O. taurus,* the closest species to *P. japonica* whose ORs have been studied in detail, is characterized by a single copy of Group 7 OR, whereas it shows a peculiar paralogous expansion of Group 6 [15]. At variance, other OR subfamilies display a sizable paralogous expansion in the JB, namely Groups 2B, 3 and 4, an expansion that is most evident in the latter, with a total of 23 ORs compared to 1 to 4 in the other species studied here (Figs. 5, 6) and elsewhere [15]. Based on the phylogenetic tree of ORs, the group 4 clade splits, with high support into two sublineages: some sequences are closely related to other beetles’ orthologs, while 13 ORs (12 genes *plus* one alternatively spliced transcript) apparently follow a different evolutionary trend. Being nevertheless associated they have been described as belonging to Group 4 (Fig. 5). Since Group 4 is considered as one of most conservative subfamily among ORs [15], and this paralogous expansion has been recorded only in *P. japonica,* we can speculate on a possible role of this latter in the evolutionary ecology of this species. The exact biological function of ORs in beetles is not fully understood, at least to the level that a single OR is associated to the sensing of a specific volatile compound [15]. Nevertheless, their role as receptors in olfactory sensory neurons, and the fact that each OR is responsible for the detection of one single molecule is proven, and this entails that the larger the OR repertoire is in a species, the larger the number of volatile compounds can be sensed and can awake a specific response by the insect. This has obvious consequences in terms of the possibilities of a species to locate and identify different sources of food and pheromones [15, 48], fostering polyphagy. In fact, the diversity of ORs has been associated to the ecological role played by a species. Overall, polyphagous beetles display a greater expansion of OR genes than oligophagous species. Similarly, free-living organisms have a larger number of ORs than parasites [15]. Therefore, it could be speculated that the great diversity of ORs observed in the genome of *P. japonica* may be connected to its polyphagous feeding habit, increasing the possibilities to exploit different plant species as food and, in turn, facilitating the expansion to different habitats characterized by a different plant diversity.

Similarly, IRs are to some extent overrepresented in the genome of *P. japonica*. The number of paralogous copies in each subfamily is in line with other coleopterans studied, but a bias can be observed towards antennal IRs (Fig. 7). Nevertheless, while in *Drosophila* species IRs are known to have a central role in gustation, temperature and humidity sensing [17], their function in beetles is still not fully understood [19]. Therefore, any speculation about the reduction of divergent IRs and the gain of multiple copies within the antennal clade in *P. japonica* may be not justified at this stage. Worth of note is a non-characterized clade of 9 IR genes (which encodes for 17 splicing isoforms) within the antennal clade, here defined as “JB basal IRs” (Figs. 7, 8). This latter appears as a synapomorphic trait of the JB, at least limited to the coleopteran species studied here. Being these sequences specific to *P. japonica,* they might be a possible object of further investigation, trying to identify their specific function, that may in turn be related to specific adaptations.

A different pattern of orthology characterizes the GR repertoire of the JB that, unlike in ORs and IRs, appears to be reduced. In terms of the overall gene number, *P. japonica* is in line with *A. planipennis* and *D. ponderosae,* but has a significantly reduced repertoire if compared to *L. decemlineata* and *T. castaneum*. Concerning the three GRs that have been better characterized in terms of function, *P. japonica* displays few copies of CO_2_ and sugar GRs genes and apparently lacks the fructose GR gene altogether (Fig. 4, Tab. 1). This is obviously at odd with the capability of *P. japonica* to feed on fruits, where both fructose and the CO_2_ produced during fruit maturation may represent relevant signals. Nevertheless, the absence of one specific receptor does not necessarily imply a general loss of function. For example, the GR for CO_2_ is lacking in the hymenopteran *A. mellifera*, while it is conserved even in species (i.e., the coleopterans *D. ponderosae* and *A. planipennis*; the dipteran *Mayetiola destructor)* that experienced a contraction of GRs comparable to that of *P. japonica* [12, 19, 64]. In fact, *A. mellifera* has been shown to be fully capable of responding to CO_2_ levels [64], leading to the hypothesis that the function of the canonical CO_2_ receptor may have been recovered through an alternative receptor [12], perhaps to be found among the large repertoire of non-characterized GRs (Fig. 4).

In the CYP 450 repertoire of *P. japonica*, the mito clan, encoding for proteins located in mitochondria, and clan 2, whose representatives are involved in multiple functions [14, 65] display a limited diversity. These two clans are also very conserved in other beetles. On the other hand, the two dominant clans in *P. japonica*, as well as in other beetles, are clan 3 and clan 4 (Figs. 9, 10). Despite their diffusion, they are, with few exceptions, poorly studied and their individual role is still unclear [14]. They have nevertheless been variously associated to key functions, from insecticide resistance to detoxification and the metabolism of foreign compounds [14, 21]. The expansion of these two gene clans in *P. japonica*, together with the expansion of specific orthologs in clans 3 and 4, is suggestive of an increased capacity of the species to cope with foreign compounds, both natural (i.e., plant-derived phytotoxins or other deterrent molecules) and human-administered (i.e., insecticides). Despite some evidences that do not prove a co-linearity between CYPome size and the ecological role played by the species (see [14] for a better overview), our results are suggestive of the fact that the expansion of *P. japonica* cytochrome P450 repertoire is related to its invasiveness. This relation rests on the capacity of the JB to a) feed on a large number of different plants, b) adapt to new ecosystems characterized by a diverse floral assemblage, and c) resist to chemical control measures (which, for example, has been extensively applied in the early days of *P. japonica* control in the USA).

## Conclusions

Notwithstanding some limitation in the current assembly (sequence arising from a pool of multiple individuals, absence of chromosome level scaffolding) we see the description of this genome as a relevant contribution to the knowledge of the JB biology, and a valuable piece of information to support the development of control/eradication methods based on genomic data. In fact, the availability of a high-quality genome, inclusive of structural and functional annotation, opens countless opportunities to further study the biology and adaptations of the species, as well as to develop biotechnological applications to control the pest or integrate molecular genetic information to steer the advancement of non-biotechnological approaches. As a preliminary example of the potential usefulness of these data, we identified a rich repertoire of genes in the genome of *P. japonica*, with significant expansion in some families, that may be associated to the staggering capacity of the species to feed over a variety of different plant species, invade new areas and resist to control measures, that may be in turn interpreted as one of the drivers of the Japanese beetles’ population development. Further implementations of this genome might rely on the use of tissue specific RNA-seq in order to detect and annotate tissue-related genes such as odorant and ionotropic receptors (mainly expressed in antennae and maxillary palps), and a manual curation that maybe nevertheless more profitably attempted once the genome reaches the chromosome-level stage.

### Supplementary Information

**Supplementary Material 1.**

**Supplementary Material 2.**

**Supplementary Material 3.**

**Supplementary Material 4.**

**Supplementary Material 5.**

**Supplementary Material 6.**

**Supplementary Material 7.**

**Supplementary Material 8.**

**Supplementary Material 9.**

**Supplementary Material 10.**

## Data Availability

All sequence data were submitted to NCBI under Bioproject PRJNA860365 as follows. Biosamples: SAMN29883562-SAMN29883565 (genome sequencing, individual samples), SAMN34257817 (mock individual, linking to the 4 aforementioned samples, associated to the final genomic sequence), SAMN29883566-SAMN29883573 (RNA sequencing). Short Read Archive (raw reads): SRR20647930, SRR20647939, SRR20647946 (genome sequencing, short reads), SRR20647937, SRR20647940 (genome sequencing, long reads), SRR20647941, SRR20647932, SRR20647934, SRR20647936, SRR20647943, SRR20647945, SRR20647948, SRR20647950 (mRNA sequencing), SRR20647931, SRR20647933, SRR20647935, SRR20647938, SRR20647942, SRR20647944, SRR20647947, SRR20647949 (total RNA sequencing). Annotated genome sequence: JASPKY000000000.

## Abbreviations

JB: Japanese Beetle
GR: Gustatory Receptor
OR: Odorant Receptor
IR: Ionotropic Receptor
CYP: Cytochrome P450
ONT: Oxford Nanopore Technology
TE: Transposable Element
LINE: Long Interspersed Nuclear Element
LTR: Long Terminal Repeat
RC: Rolling Circles
