# Supplementary figures and images for: De novo assembly and annotation of Popillia japonica’s genome with initial clues to its potential as an invasive pest
Source: BMC Genomics. 2024 Mar 13;25:275. doi: 10.1186/s12864-024-10180-x (PMC10936072; doi:10.1186/s12864-024-10180-x)

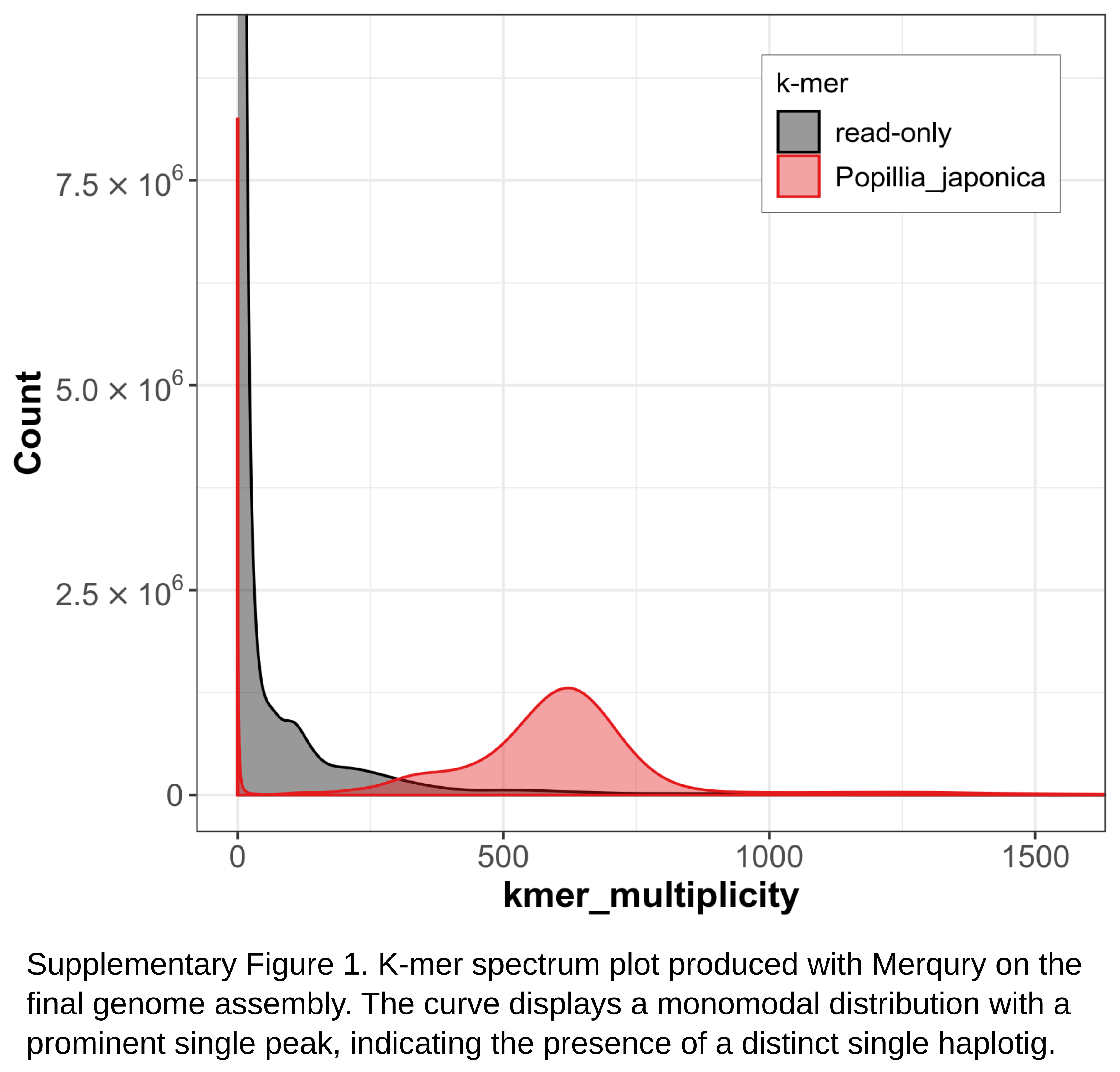

Supplement: Supplementary file 1 — Supplementary Material 1. [file 12864_2024_10180_MOESM1_ESM.png]

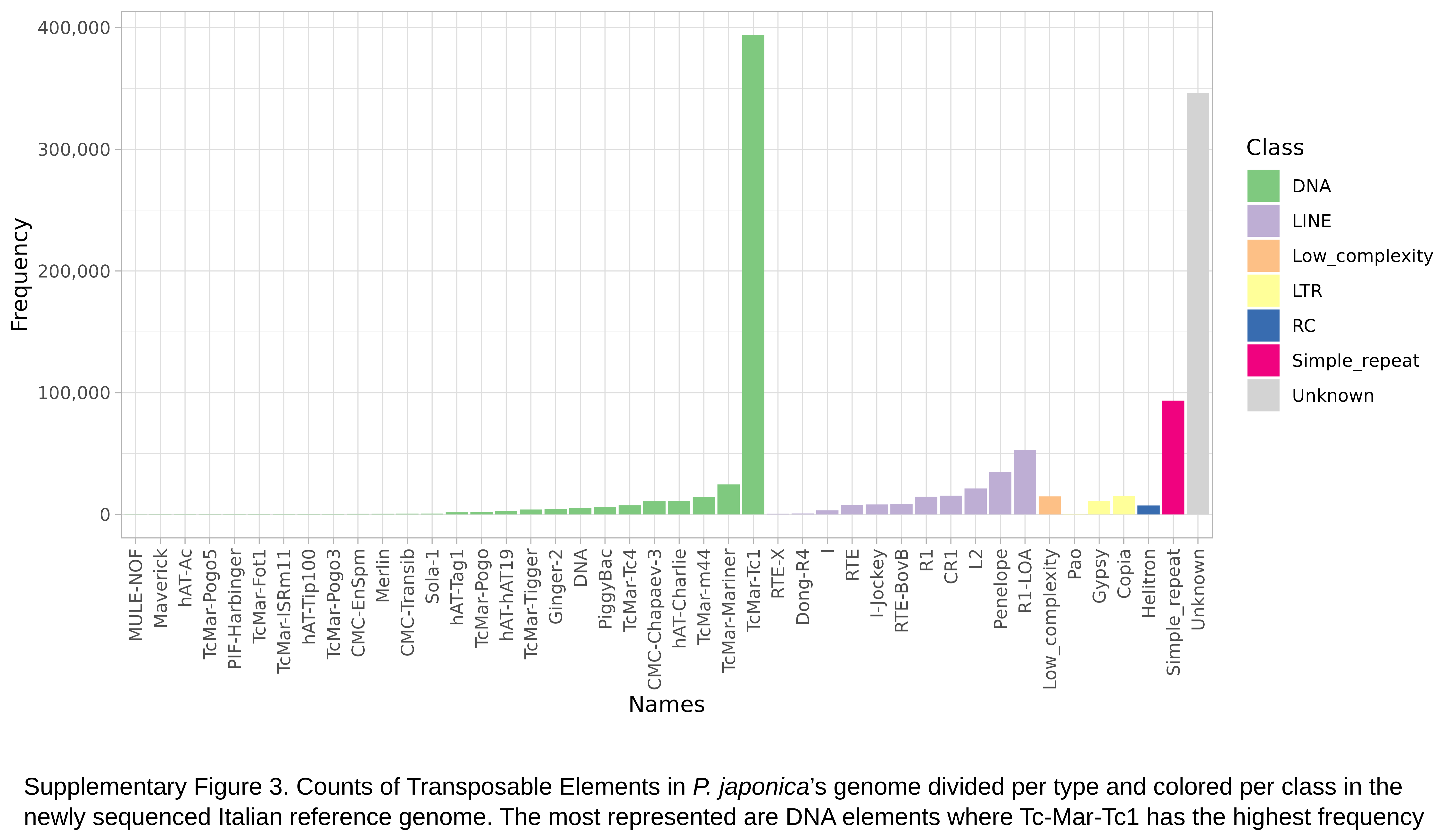

Supplement: Supplementary file 3 — Supplementary Material 3. [file 12864_2024_10180_MOESM3_ESM.png]

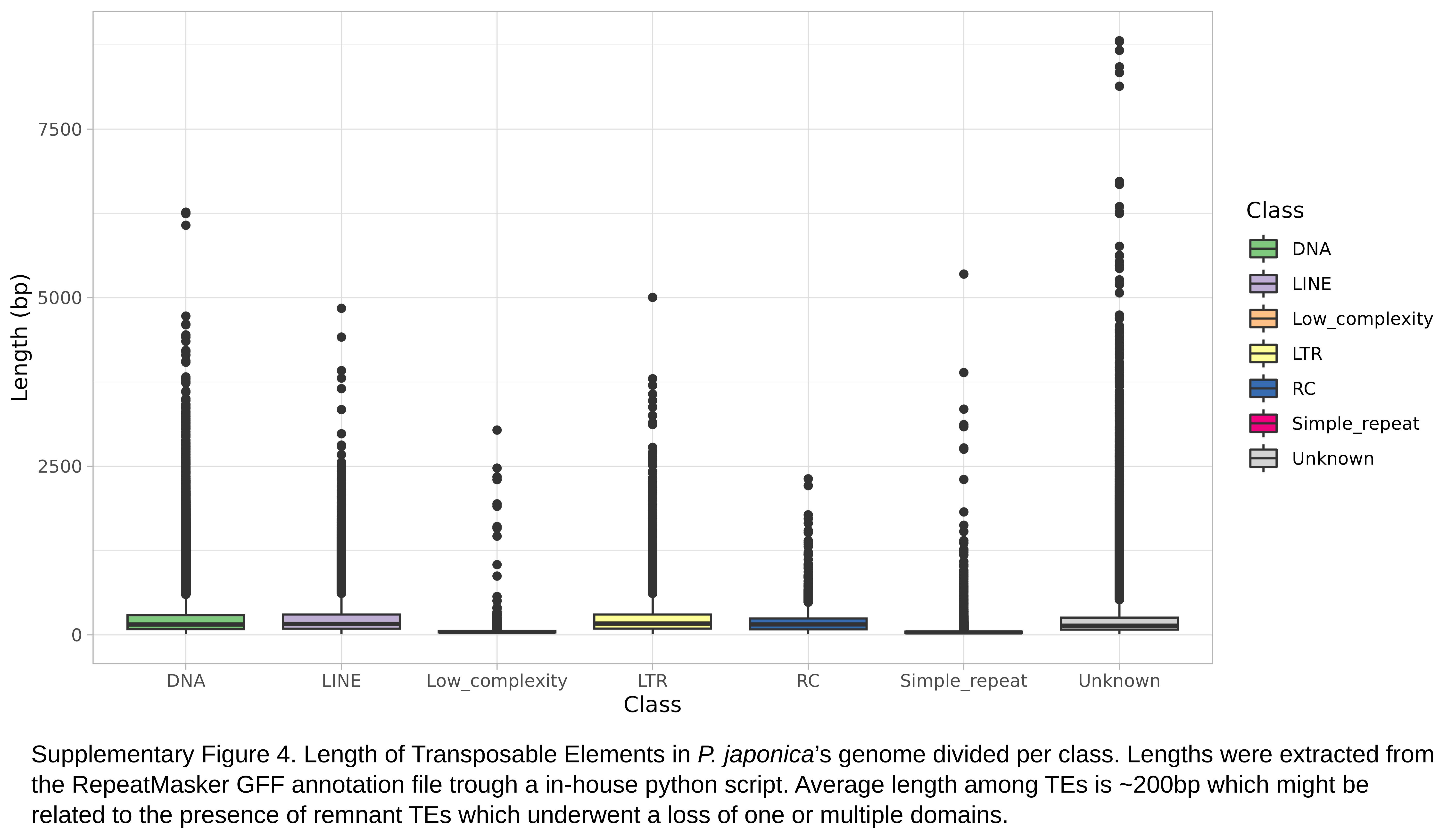

Supplement: Supplementary file 4 — Supplementary Material 4. [file 12864_2024_10180_MOESM4_ESM.png]

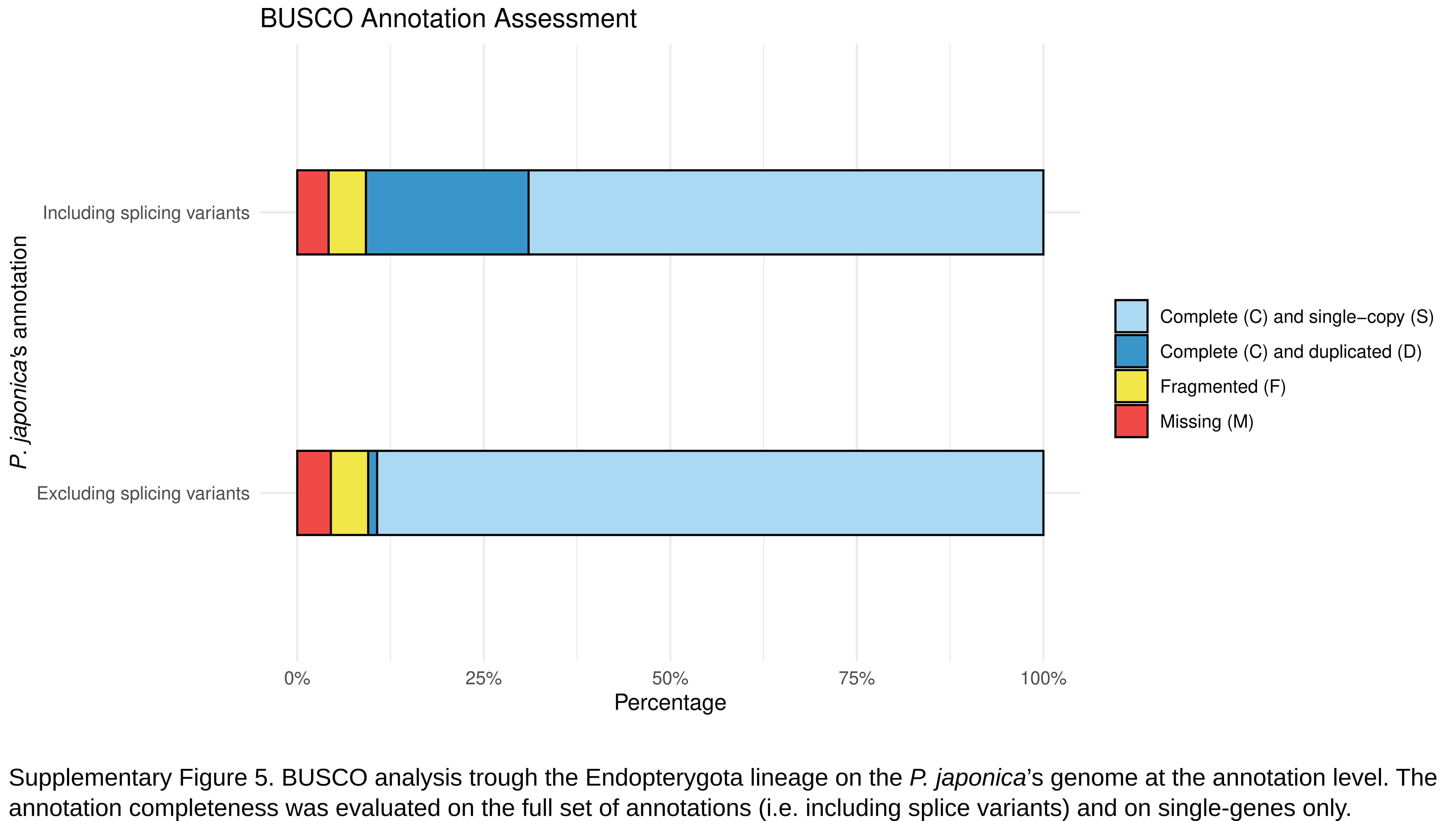

Supplement: Supplementary file 5 — Supplementary Material 5. [file 12864_2024_10180_MOESM5_ESM.png]

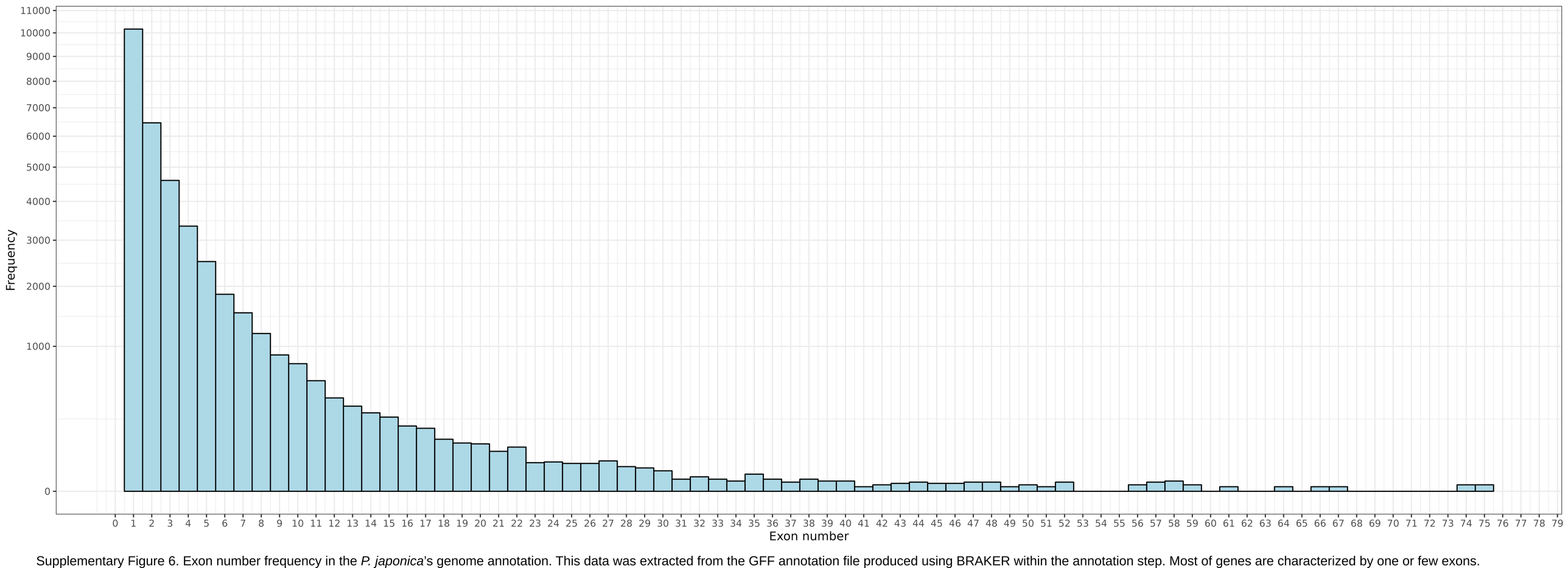

Supplement: Supplementary file 6 — Supplementary Material 6. [file 12864_2024_10180_MOESM6_ESM.png]

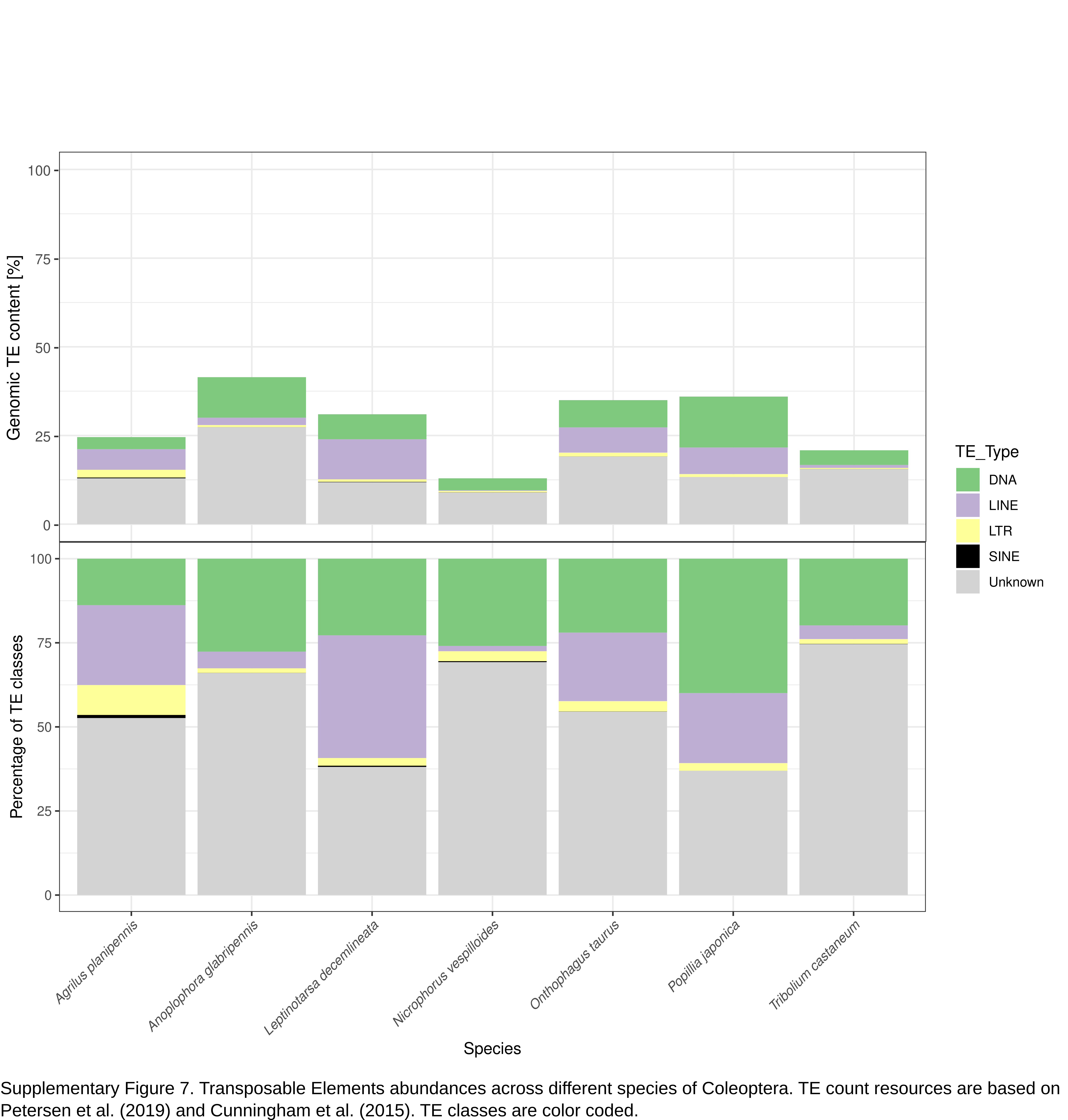

Supplement: Supplementary file 7 — Supplementary Material 7. [file 12864_2024_10180_MOESM7_ESM.png]
