# Supplementary material for: De novo assembly and annotation of Popillia japonica’s genome with initial clues to its potential as an invasive pest
Source: BMC Genomics. 2024 Mar 13;25:275. doi: 10.1186/s12864-024-10180-x (PMC10936072; doi:10.1186/s12864-024-10180-x)
Supplement: Supplementary file 2 — Supplementary Material 2. [file 12864_2024_10180_MOESM2_ESM.pdf]

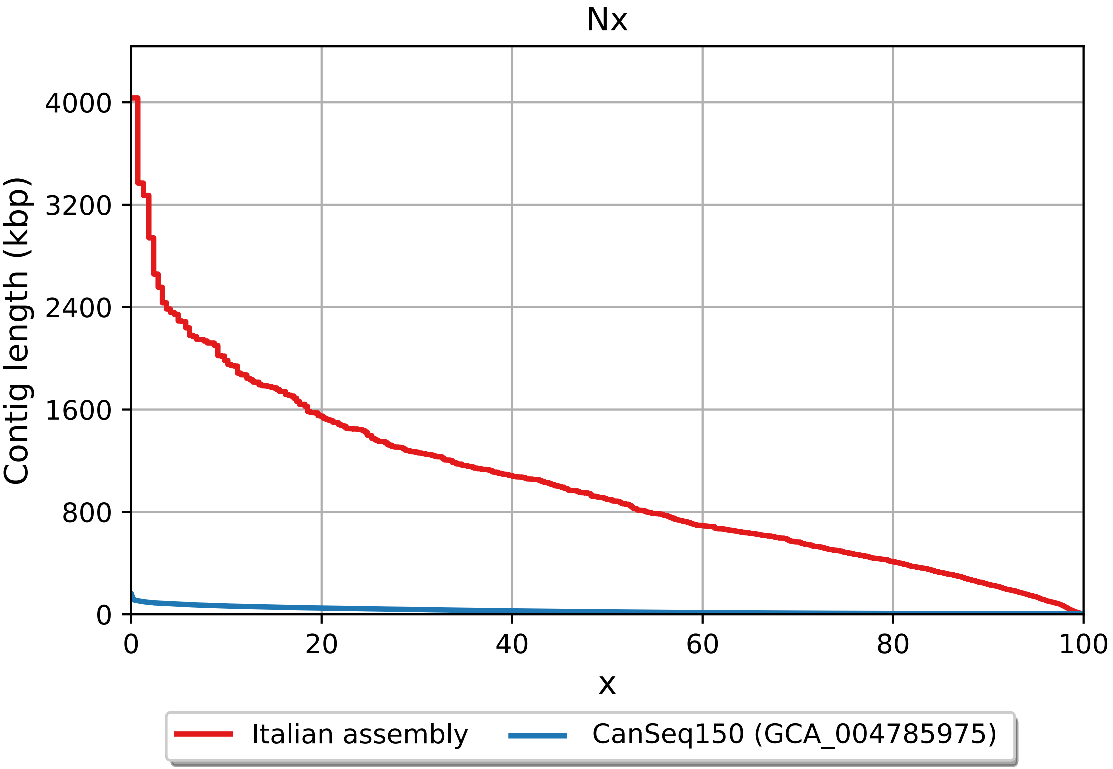

Supplementary Figure 2. QUAST-LG Nx comparison between the Italian genome and the CanSeq150 Canadian genome. The contiguity of the new Italian assembly is extremely higher than the CanSeq150 genome.
